# Cause and Cure of Disease

**Published:** 1888-03

**Authors:** 


### "CAUSE AND CURE OF DISEASE."

Progress or the law of higher and more perfect conditions is the tendency of all things; and all things would follow that law; but antagonistic forces prevent.

All nature is an exhibition of the two forces, one towards harmony from chaos; the other, to chaos and inharmony; one gives health and beauty; the other dwarfs and diseases.

Progress is seen in the knowledge of man, physiologically, anatomically and hygyenically. Greater skill exists now than in the past by which the surgeon can perform feats that are indeed marvelous, upon the bodies of the diseased.

Mechanical appliances and apparatus for the deformities, which have come about by nature's harmony being interrupted, are nicely adjusted to the ends for which they were made.

Sanitary science has made great strides, towards erecting healthy conditions where disease dwelt and reigned supreme.

Health publications, in the way of books and magazines, are disseminated among the masses of unintelligent as well as intelligent, dissipating ignorance from the minds with reference to man and his relations to the things he eats, the air he breathes, the clothes he wears, the habits he forms, and the associations which surround him, as to whether these things are useful or injurious.

In medicine, the law of progress has been and is checked by which dwarfing states exist, showing a tree, whose fruitage is one of bitterness and death to all who eat of it, any great length of time.

Medicine is called a science by those who use it for a business, but it only requires a small portion of learning and observation to show that the claim is false.

Why does medicine exhibit such poor progress towards scientific results when applied? What disease is, has not yet been accurately defined, as no two celebrated physicians will agree in the diagnosis of a case.

To destroy an enemy and not know where to strike, nor what weapons to use, as the experimentations of all medical systems have done for centuries, can not be called very scientific.

The "germ theory" of disease is the latest deduction and supposed cause of disease; it is not shown clearly from whence the germ comes, nor how they get into the human organism, all is hypothesis.

What medicines are and what they do, whether good or injury to the organism is all conjecture, in so far as absolute facts are concerned, from the medical theology point of view.

One often gets well after swallowing drugs; but it is not known that the drugs produced the wellness, as many get well without the use of drugs at all.

Disease exists as a fact; all are more or less living exponents of the truth of the statement; and centuries have swept by, in which human efforts have been put forth to check the ravages and death, which are produced by it.

Victory has more often been on the side of disease, even when all the learning, all the skill and all the remedies known, have been applied. Victory has often been exhibited, when the *practitioner* was defective of learning in *medical* lore or medical skill, or experimentation.

Confidence in the power of drugs to cure, has been lost, because of the facts of wonderful cures being performed by the *unlearned* and wonderful cures being effected without the use of drugs.

Bold reformers have arisen, all along the line of human civilization, opposing the various methods and systems of cure, thereby establishing *new systems*, new definitions, and many *sects* in medicine; who often hate each other with the same hatred, as formerly existed among the various religious sects.

*Hygiene* is a result of reform, opposing the use of drugs and going to nature for relief. The boldest and most powerful champion the world has produced in the cause of "Hygiene *vs.* Drug Medication," was Dr. Trall, whose name will yet be given due recognition for his labors in behalf of truth as he saw it.

Another bold and powerful champion comes to the front for a hearing, with a volume, entitled "Cause and Cure of Disease," in which will be found *great light* on old subjects; subjects which none of us were conscious, had any bearings upon causes and cures of diseases of the human family, also, what effects, mineral medicines have upon the organization, whether injurious or beneficial. There is no difference between a medicine and a food. If a so-called medicine is taken internally and does not produce the same results that a food does, it is injurious to be taken. These definitions of disease, of medicine and the methods of cure for all curable diseases, which are not diseases at all, evidently, place a scientific value upon a knowledge of medicines and disease and its causes, which the whole medical literature of the past has not done heretofore.

I call the attention of the reader, to this subject because I desire that more knowledge, and thereby more health shall come into the world and lift us all into a higher conception of the reign of *Harmony* and *Love* on earth.

DELAWARE, O., January, 1888. L. EMERICK.

---

*To the Editor of* HALL'S JOURNAL OF HEALTH:

DEAR SIR—A few days after your estimable JOURNAL was put in circulation, I received by mail, a communication in the shape of an extract of the "Bureau of Record" (medical) demonstrating the scientific necessity of total abstinence. Considering this message to be a reminder of the unscientific spirit I seemed to breathe into my article on "Temperenzia," I feel in honor bound to enlighten my reader on the real drift of my article in question.

I am well familiar with the toxical and physiological effects of the alcoholic poison, and, in my limited sphere of professional usefulness, I seized on every occasion to inculcate the necessity of temperance in all things, but

I did not waste my time in preaching an ideal tetotalism to people who seem to have long ago relegated all pretentions of an ideal life to the Sunday pulpits, in order to keep the clergy occupied.

Intemperance seems really to be the cankerous diathesis of our modern civilization all over Christendom. The medical profession itself, seems to be infected, to a great extent, by the evil of intemperance in their hasty application of poisoning narcotics, and stimulants of all kinds in various ways. Even their resources to instrumental application, escharotics and other less destroying irritants is marked in a great many cases, by intemperate habits. Intemperance seems, also, to have invaded the legitimate domain of bodily exercise, which has degenerated into the promotion of brutality in the shape of speculative pugilism and gluttony, for the benefit of our gambling institutions. The betting on how many dozens of eggs and the number of dishes a certain expert could swallow in one meal, has filled many a gambler's pocket. But the contagion of intemperance is marked even in our educational institutions. If you observe our happy youth going home from school loaded with books on their arms, the weight of the load corresponding, it is believed, to the degree of knowledge acquired, you guess at once the overstrain put on the nervous system of our tender youth for very little purpose. A smattering of many things and a poor ephemeral knowledge of any of the objects crammed in their heads. Hence, you meet many a youth leaving school with credentials yet scarcely able to clothe their shooting ideas in decent and correct language. You see that alcohol is not the monopoly of intemperance, but certain it is that alcoholic intemperance can never be successfully counteracted, by the formalism of pledge taking of total abstinence, preceded by insignificant exhortation of our popular lecturers. Temperance in all things, as the true spirit of the Nazarene ethics, should be the *principal* theme of our pulpit oratory, hammered at Sunday after Sunday, and on all occasions, while persons familiar with toxic and the physiological effect of narcotics, should be employed, on week days, to enlighten the ignorant about the danger of alcoholic beverages in particular, which measures have a better prospect of real success.

B. L. C.

---

The philosophy of one century is the common sense of the next. We should so live and labor in our time that what came to us as seed, may go to the next generation as blossom, and that what came to us a blossom may go to them as fruit.—*Henry Ward Beecher.*

